# Chicago Gynecological Society

**Published:** 1898-11

**Authors:** 


### *SOCIETY REPORTS.*

### CHICAGO GYNECOLOGICAL SOCIETY.

*Regular meeting held September 18, 1898.*

The President, Dr. Henry P. Newman, in the chair.

Dr. Joseph Price, of Philadelphia (by invitation), read a paper entitled "Abdominal *vs.* Vaginal Section in Pelvic Surgery."

He said it was very difficult to consider some of the opinions of recent adoption by surgeons with a judicial temper and impartiality. These different opinions had a surgical importance, in that problems arose, and their discussion would not and should not down until they were satisfactorily settled. If his own views were distinct and had something of a dogmatic ring, it was because they were forced upon him by actual experience and observation. Many practising the vaginal operation had the impression that the operation had not been universally adopted because physicians did not understand it; that it was difficult, dangerous, or impossible in their hands; that it required for its successful performance a peculiar aptitude, a special training and adeptness. This was a mistake. A number of men who opposed the vaginal operation had done it successfully; their mortality had been quite as low as that of those who advocated and made the procedure their adopted one. They did the suprapubic operation, influenced by the logic of their experience, by purely surgical and pathologic reasons, and it was the operation of their choice because it gave the most complete results, left less dangerous or annoying sequelæ, and less risk of the necessity for repeated operations. By the suprapubic route surgical cleanliness and surgical completeness were possible; by the vaginal they were not. The difference between the advocates of the abdominal method and those who criticized it was that the advocate spoke according to his knowledge and of the facts which actual clinical experience had confirmed; the critic according to his failures, disappointments and prejudices. The one had uniform success sustaining him; the other humiliating failures which inspired and gave color to his opinions. The French and Belgians were never successful in doing the suprapubic operation. The successful American, English and German operators had practised both methods for years, adopting the suprapubic procedure for tubal and ovarian disease, and the vaginal route for malignancy. The results of these men were uniformly good; they adapted their operation to actual pathological conditions; they operated for actual disease, and not for all sorts of fancied conditions, or for vague nervous disorders due frequently to emotional susceptibility.

He said it was simply amazing how common it was with some operators to begin two distinct operations and complete neither. Good operators sometimes made a free opening into the abdomen, inspected and backed out, and then attempted the vaginal route and abandoned it after the puncture of one or more accumulations and drainage. The relief following mere puncture and drainage was only temporary. The drainage of one or more pus-pockets, where many existed in a large, tortuous, puriform tube or tubes, and where we had one or more ovarian abscesses, never cured. The only cure was the removal of the diseased member. He thought surgeons need only appeal to actual clinical facts in the experience of some prominent vaginal operators to show how difficult and incomplete their work was when they encountered deep-seated and complicated pathologic conditions, and also in how very many cases the result was fatal. In relation to results from any procedure, no reliance could be placed on the statistics of men who selected only favorable cases for operation and rejected the unfavorable. These men in his opinion had no right to compare their results with those of men who did not reject the desperate cases. Imperfect and incomplete work by the abdominal route was a feeble argument in favor of the vaginal. The fault was not in the procedure, but in the operator, his lack of wide clinical experience in dealing with gynecologic troubles or lack of surgical courage to complete the work he began. The removal of pathologic conditions was easier from above than from below, because the structures were more easily defined and lines of cleavage or enucleation were *from* important structures and not *toward* them or *into* them; there was no difficulty in securing arteries, as they could be seen and felt pulsating beneath the fingers. The operation was precise; it could be made mathematically certain in its limits; the incision was directly under the eye and under the absolute control of the fingers; it was not a stab about in the dark among vital organs as in the vaginal route; it enabled the easy freeing of omentum and bowel when adherent, and the repair of all disorganized parts. These were important considerations.

Careful examination of statistics coming from reliable sources went to show that abdominal pain continued in very many cases operated upon by the vaginal route and followed too many imperfectly, incompletely and ignorantly operated upon by the suprapubic method. These disagreeable symptoms complained of by patients after operation were nearly always the result of leaving omental and intestinal adhesions. The profession was too prone to talk about the septic uterus. A patient with a septic uterus was very ill and usually died speedily. There were few things that killed a woman quicker than a septic uterus. The essayist was daily doing sections, and while dealing with all sorts of complications and adhesions, dangerous twists and contortions, strong adhesions or fixation of crossed viscera, the sigmoid strongly adherent to the anterior face of the right tube and broad ligament, the cecum and appendix out of position and adherent, he could not but experience a sense of surprise that experienced surgeons (who have in the past done good abdominal work) could forget or ignore the lessons of their experience and deliberately extirpate the little healthy uterus and pass by pathologic lesions and complications constituting the real and only source of trouble. Surgery would be more judicious and successful if more care and skill were exercised in determining definitely the trouble for which operations were performed and the procedure strictly adapted to actual conditions. No one method should be pursued in all cases; the symptoms and conditions present must largely guide the surgeon in the selection of a procedure. It was important to select with great care the cases favorable to the application of any particular method. Sinuses were just as frequent and distressing in the vaginal vault as in the abdominal incision. Menopause nervous phenomena were about the same in both procedures, when completed.

In a series of four hundred and three cases of vaginal hysterectomy, including about all conditions for which it was done, total general prolapse, etc., Jacobs had nine fistulæ after the operations, yet he said that "subsequent fistulæ were exceedingly rare." He had observed five intestinal, three visceral and one ureteral fistulæ. Further, he remarks that "in most of the cases these fistulæ existed prior to the operation; there were fistulous passages which extended between the purulent foci and some part of the intestine. These passages were so large, and with walls so well organized that the disappearance of the purulent pockets did not suffice to bring about the subsequent and spontaneous cure." In all such cases the speaker relieves adhesions, trims and repairs all lesions, with the most pleasing results, without any of the sequelæ of fistulous openings given by Jacobs as following the vaginal procedure.

Dr. Fernand Henrotin opened the discussion with a paper entitled "The Indications for Interference by Way of the Vagina in Pelvic Diseases: An Answer to Dr. Joseph Price."

At the outset, he held that the vagina route was a proper channel to attack pelvic disease in women in certain selected cases only. As better inspection and palpation of the pelvic organs could be obtained by an abdominal incision, it was conceded that all patients to be operated vaginally must present special indications, and that in cases of doubt the abdominal incision was most proper. In favor of vaginal section it may be said that where the same results can be obtained vaginally, this route should be accepted, as it avoids the abdominal scar, lessens the shock and is much less frequently followed by hernia. Although one of the first to advocate the treatment of pelvic affections by way of the vagina in selected cases, he was most willing to admit that this method was not so much in vogue with experienced operators as it was three years ago, and for very good reasons, one of which was that our knowledge of the prevalence of appendicitis in conjunction with pelvic trouble made it incumbent to operate by an abdominal incision when any degree of this affection was even suspected, and we had learned in later years that this condition was common. The primary incision, whether it be through the abdomen above the pelvis, or through the vaginal vault, was to a degree always an exploration. The infinite variety of intra-abdominal complications made it so. This primary incision was as frequently curative by way of the vagina, and even more frequently so, than the abdominal cut. This was by reason of the ease and directness of drainage. The early vaginal incision, to those who understood it and had the skill to perform it properly, was the ideal of conservative surgery. This incision, like all vaginal operations, was only applicable to selected cases. By early vaginal incision was meant an incision that cures the localized septic pelvic infection in its very beginning, the woman remaining thereafter not only symptomatically, but physiologically perfect. It was particularly applicable to the treatment of acute ovarian abscess. This disease, he believes, is more common by far than is generally supposed. Its most general cause was abortion and trauma. It was the most common extra-uterine form of pelvic sepsis that occurred following early miscarriages, criminal abortion and unclean surgical manipulations. After two or three days of fever the presence of exudate could usually be recognized at the sides or behind the uterus. This meant ovarian abscesses in eight or nine cases out of ten.

Vaginal hysterectomy was sometimes advisable, and in some cases was infinitely superior to any abdominal operation that could be performed on the same patient. Experienced operators who were equally skilled in vaginal, as well as in abdominal work, were doing less vaginal work now than they were two years ago. At least, this was his belief; not because vaginal hysterectomy was not a very proper and sometimes the best operation to be performed in certain cases, but because it was a radical operation and because the field of all radical operations had been much restricted for the last few years. Salpingectomy, ovarian resection, vaginal incision, had all done their share in the salvation of scores of uteri. While abdominal operators were improving their methods and perfecting their results, the vaginal workers were developing the possibilities of the vaginal incision, and he claims to-day that one, if not the greatest of modern conservative gynecologic triumphs, is the thoroughly understood and properly performed vaginal section in selected cases. And there are many. As regards vaginal hysterectomy, it still had, in the opinion of the speaker, a perfectly defined position. In women necessarily sterile and approaching the menopause, where bilateral periuterine septic disease existed, and was situated low in the pelvis and with a roomy vagina, particularly in those with extensive and disseminated suppuration, who were low with septic fever, and especially where abdominal wall was very fat, vaginal hysterectomy was still by far the preferable operation, and when skillfully performed, in a large series would always give the best results. To demonstrate his own opinion as regards selection, Dr. Henrotin presented a table showing the frequency of vaginal as compared with abdominal incision, and the character of the intervention, from which it will be seen what an important factor the conservative operation has become, and frequently he believes vaginal section applicable. These operations were performed from January, 1897, to July, 1898. Reference is made only to such as were done for clearly defined pelvic disease, and the list does not include abdominal operations on the kidney, liver, gall-bladder, appendix, intestine (hernia), or any miscellaneous intra-abdominal work in which the internal genitalia were not involved. Of 180 such operations there were :

SUPRAPUBIC CELIOTOMIES, 142.

| | |
|---|---|
| Conservative operations on the adnexæ, always leaving ovarian tissues and uterus | 62 |
| Double salpingo-oöphorectomy | 4 |
| Single salpingo-oöphorectomy for ruptured ectopic gestation | 8 |
| Myomectomy | 3 |
| Hysterectomy for fibroids | 14 |
| Hysterectomy for sepsis | 8 |
| Hysterectomy for prolapse | 3 |
| Miscellaneous operations for adhesions; ovarian, dermoid and intra-ligamentous cysts; sarcomas and hard ovarian tumors, etc | 30 |
| | 132 |

VAGINAL OPERATIONS, 44.

| | |
|---|---|
| Early section for recent disease | 8 |
| Late section for old disease | 18 |
| Single salpingo-oöphorectomy | 1 |
| Section for large ovarian cyst, and its removal after tapping | 1 |
| Section for septic ruptured ectopic gestation | 1 |
| Hysterectomy for sepsis | 10 |
| Hysterectomy for fibroids | 2 |
| Hysterectomy for cancer | 1 |
| Hysterectomy for prolapse | 2 |
| | 44 |

Vagino-abdominal operations for cancer, 4. Total, 180.

Dr. Franklin H. Martin said that the best all-round gynecologist was the one who selected the operation suitable for the particular case in hand. It was impossible to do good surgery by operating suprapubically or by the vagina in all cases. It was his belief that all major surgery could be done best through the abdomen, as a rule, while all minor surgery could be done by the vagina. All surgery of importance should be done through the abdomen: 1, because the surgeon could see what he is doing; 2, he could do what he wanted; and 3, he could do it quicker. The cases he would reserve for the vaginal route would be those of carcinoma of the cervix of the proliferating variety, or flat cell epithelioma filling up the vagina, but which seldom extended beyond the cervix and infected the lymphatic glands. In all other cases, where there was any question as to cancer involving the vagina, he would remove the uterus from above, and if later it was found that the glands were diseased, he would remove them also. Cases of double pyosalpinx, large fibroids, cystomata and all cases of extra-uterine pregnancy should be dealt with supraupbically.

Dr. Henry T. Byford held that to operate on all cases either suprapubically or by the vagina was unreasonable. He believed that just as efficient work could be accomplished by the vagina as through the abdomen. There were certain pathologic conditions in the bottom of the pelvis in some cases that could not be operated as well from above as from below. He emphasized the importance of carefully selecting the cases for either route.

Dr. James H. Etheridge thought the discussion had the appearanee of placing the advocates of the two operations in the attitude of rivals. He did not believe this. He held that we have two separate things to deal with, and that good work could be done by the vaginal or suprapubic method of operating. There were cases in which the vaginal route was applicable, while there were others in which the abdominal method was suitable, and the wisdom and experience of such men as Drs. Price and Henrotin could define the limits of those cases to be operated upon vaginally and those that were best adapted to the suprapubic route. A strong objection to the vaginal route was that operators worked largely in the dark. Hemostasis was unsatisfactory and, added to this, there was danger of wounding the bowel. He had seen one case in which death was caused from wounding the bowel, but which was only discovered post mortem.

Dr. Reuben Peterson said that any one who had done considerable abdominal work must have seen cases that he dreaded to approach by the suprapubic route. For many years nearly all work was directed entirely through the abdomen in dealing with the pathological conditions under discussion. Each method had its field of usefulness, and it remained for gynecologists to select their cases and choose the method to employ.

Further discussion of this subject was deferred until the next meeting.

Dr. Emil Ries read an inaugural thesis entitled "Results of the Extended Operation for Carcinoma Uteri."—*Journal of American Medical Association.*

---

### FOR SYPHILITIC ARTHRITIS.

In cases in which the acute local pain and sensitiveness preclude mercurial inunctions or subcutaneous injections, it is recommended to begin with internal mixed treatment as follows:

| | | |
|---|---|---|
| ℞ | Hydrarg. iodi rubri | gr. iss. |
| | Potassii iodi | gr. lxxx. |
| | Sherry wine | ʒ v. |
| | Aquæ q. s. ad. | ℥ vi. |

M. Sig. Two tablespoonfuls a day.—*Ex.*

---

### FOR PSORIASIS.

The following internal treatment is recommended by Brorý in conjunction with external applications:

| | | |
|---|---|---|
| ℞ | Sodii arseniat | gr. $\frac{1}{10}$. |
| | Sodii salicylat | gr. xlv. |
| | Sodii bicarb | ʒ ii. |
| | Syr. saponariæ | ℥ iv. |
| | Tinct. gentian. comp. }<br>Syr. simplicis } aa | ℥ ii. |

M. Sig. One tablespoonful three times a day at mealtime.—*Ex.*

